# BDS Dual-Frequency Carrier Phase Multipath Hemispherical Map Model and Its Application in Real-Time Deformation Monitoring

**DOI:** 10.3390/s23146357

**Published:** 2023-07-13

**Authors:** Ao Sun, Qiuzhao Zhang, Xingwang Gao, Xiaolin Meng, Yunlong Zhang, Craig Hancock

**Affiliations:** 1 School of Environmental and Spatial Informatics, China University of Mining and Technology, Xuzhou 221116, China; ts21160021a31ld@cumt.end.cn (A.S.); ts19160093p31tm@cumt.edu.cn (X.G.); 2 College of Architecture and Civil Engineering, Beijing University of Technology, Beijing 100124, China; mengxl@bjut.edu.cn; 3 National Engineering Laboratory for Digital Construction and Evaluation of Urban Rail Transit, China Railway Design Group Co., Ltd., Tianjin 300308, China; zhangyunlong@crdc.com; 4 School of Architecture, Building and Civil Engineering, Loughborough University, Leicestershire LE11 3TU, UK; c.m.hancock@lboro.ac.uk

**Keywords:** multipath, BDS, single difference residuals, multipath hemispherical map, deformation monitoring

## Abstract

The BDS multipath delay error is highly related to the surrounding monitoring environment, which cannot be eliminated or mitigated by applying the double difference observation model. In the actual monitoring environment, due to the complexity of the BDS constellation, it is difficult for existing algorithms to consider GEO, IGSO, MEO and other different orbital types of satellites for real-time and efficient multipath error reduction. Therefore, we propose a novel BDS dual-frequency multipath error reduction method for real deformation monitoring for BDS considering various satellite orbit types. This method extracts the single error residual of each satellite based on the assumption of “zero mean” and divides the appropriate grid density of GEO and IGSO/MEO, respectively, to construct a dual-frequency multipath hemispherical map model suitable for BDS satellites with different orbital types. This method can realize the multipath error elimination of the observed values of different orbits and different frequencies. The results of simulation experiments and real deformation monitoring data demonstrate that this method can effectively eliminate low-frequency multipath delay errors in the observation domain and coordinate domain. After multipath correction, the precision of the horizontal coordinates and height coordinates are 1.7 mm and 4.6 mm. The precision of the horizontal coordinate and height coordinate is increased by 50% and 60%, respectively. The fixed rate of ambiguity increased by 5–7%.

## 1. Introduction

Beidou Navigation Satellite System (BDS) has completed the “three step” strategy [1,2]. Now, BDS can provide real-time, all-weather, high accuracy services for global users [3,4]. Moreover, the BDS positioning accuracy is equivalent to or even better than GPS and has a faster average convergence speed than GPS [5] in the Asian-Pacific region achieved by collecting more satellite data. This gives BDS advantages for deformation monitoring applications.

Recently, the application of Global Navigation Satellite System (GNSS) technology in deformation monitoring has become an important means to monitor the structural health of buildings due to its advantages in automation, its real-time and large-scale applications and its ability to operate in all weather conditions [6]. However, there are still many errors, which limit GNSS in many high-precision positioning services [3,7]. The short baseline BDS Double-Difference (DD) positioning mode can differentially eliminate the satellite clock error and receiver clock error and can significantly mitigate the high spatial correlation errors caused by tropospheric delay and ionospheric delay; however, the multipath error cannot be eliminated by DD mode and becomes the main error source in short baseline relative positioning.

BDS has three different orbit types: the geostationary orbit (GEO), the inclined geostationary orbit (IGSO) and the medium Earth orbit (MEO) [8]. Using the temporal and spatial repeatability of the multipath effect to mitigate the multipath effect in the carrier phase observations of BDS, it is necessary to take into account the small operating range of the BDS GEO satellite and the 7-day revisit period of the BDS MEO satellite, which is also the focus of this paper. Therefore, the mitigation of multipath error is more complicated than single-orbit-type GNSS systems such as GPS and Galileo [9].

Over nearly 30 years, many GNSS multipath mitigation methods have been developed which can be classified into three main branches [3], but none of these methods take into account the differences between BDS satellites of different orbital types. The first one is site selection. In this method, multipath is mitigated purely by selecting a site with minimal reflections and obstructions. However, in complex monitoring environments such as DAMS and slopes, the monitoring stations are affected by surrounding vegetation and water bodies, and the multipath effect is serious, which limits the accuracy of GNSS deformation monitoring. This method is limited by objective conditions in deformation monitoring [10]. The second method is hardware devices improvement. This method is implemented in GNSS receivers and attempts to remove multipath-affected signals. The influence of multipath is mainly suppressed by improving the antenna [11,12,13] and receiver [14,15,16] and by optimizing receiver configuration [17,18]. However, theoretically, it is impossible to completely shield the multipath signal while acquiring the GNSS direct signal.

The last and most common method is data post-processing approaches, including weight adjustment based on data signal to noise ratio (SNR) techniques [7,19]; ray-tracing approaches [10]; and sidereal filtering (SF) based on the coordinate domain [11,12] or observation domain [13,14] method, which is the most popular one.

Careful site selection can reduce the impact of multipath error but is limited in its usefulness [10]. To date, much of the research focus on mitigating multipath error concentrates on hardware or software solutions. Axelrad et al. [20] found that the orbital repeat periods of GPS satellites are not strictly a sidereal day (about 23 h and 56 min) and there is a time shift which is not a constant for different satellites. Therefore, sidereal filtering (SF) based on the observation domain, which calculates the orbital period of each satellite and extracts and corrects the multipath model on corresponding satellite observations between the observation sequences of two multipath periods, was proposed and developed. Zhong et al. [21] proposed an adaptive wavelet transformation based on a cross-validation method to mitigate GPS multipath effects. Zhong et al. [22] developed a SF based on single difference (SD) residuals for mitigating GPS multipath effects over short baselines. Dai et al. applied empirical mode decomposition (EMD) in denoising coordinate sequences of short GPS baselines [23]. Ansari and Bae proposed a wavelet and power spectrum analysis method for GNSS multipath error modeling and mitigation [24].

For BDS multipath error, Ye et al. studied the multipath repeat cycle of BDS satellites, and proposed a SF carrier phase multipath elimination approach of BDS system [25]. A multipath model extraction method based on a Kalman filter and Rauch–Tung–Striebel Smoother (RTSS) was proposed to separate the SD multipath error of BDS satellites [26]. A wavelet analysis method can achieve the same effect as this method, but it is more convenient.

The SF method faced a computational challenge to precisely calculate the orbit repeat time of each satellite at the end of multipath model for the BDS system with a heterogeneous constellation. Fuhrmann proposed a multipath stacking method based on the repeatability of multipath space. This method divides the grid into equal area, which can also effectively mitigate the multipath effect [27]. However, according to AIC criterion, the equal interval method has a better balance between computational complexity and optimal health than this method [28]. Dong et al. [29] established a multipath hemispherical map (MHM) model to achieve real-time resolution and correction of multipath errors. This method built the direct relationship between the satellite position and the multipath when the antenna and reflector environment did not change significantly. Therefore, the MHM method has been improved and expanded in various studies. Dai et al. [23] compared the MHM algorithm with the sidereal filtering algorithm and proposed an improved multipath error parameterization model for BDS GEO multipath elimination. Wang [28] introduced the trend-surface MHM modeling method (T-MHM) to fit the spatial distribution of the multipath within the grid. This was then used to alleviate the high-frequency and low-frequency multipath of precise point positioning (PPP) [30]. Zheng et al. also utilized the MHM method to mitigate multipath effects and significantly improve the positioning accuracy of PPP [31]. Ma et al. extended the T-MHM method to the field of indoor positioning [32]. Liu et al. [9] proposed a single-difference model based on the multipath hemispherical map to mitigate the BDS-2/BDS-3 multipath in a short baseline. Tao et al. [33] proposed a time-frequency mask and convolutional neural network to separate and mitigate the GNSS multipath error. Zou et al. [34] compared the performance of the multi-point hemispherical grid model (MHGM) with SF from a multi-GNSS point of view and summarized the determination method for GNSS satellites’ orbit repeat periods. The above software methods based on the spatio-temporal repetition characteristics of the multipath effect are only used to study the GPS multipath effect. If the above methods are directly applied to mitigate the BDS multipath effect without comprehensive consideration of the BDS GEO satellite and BDS MEO satellite, it may be difficult to achieve the expected correction effect. In addition, some scholars have established functional models to estimate multipath delay. Hu et al. proposed a random walk multipath mitigation method, but its effectiveness in BDS still needs to be verified [35]. Tian et al. used the least squares method to mitigate multipath errors and improve positioning accuracy [36], but this type of method may cause multipath over parameters.

However, the GNSS deformation monitoring environment is complicated, and GNSS signals are reflected by surrounding water bodies and vegetation, resulting in serious interference on direct GNSS signals, which greatly affects the accuracy of GNSS deformation monitoring. It is not universal to verify the multipath mitigating method only through simulation experiments. The current research of the MHM model mostly uses simulation experiments to verify the performance, and there are few experiments based on the real deformation monitoring situation. This study attempts to fill this research gap using two groups of experiments, including a simulation deformation monitoring scene and a real river culvert deformation monitoring scene. These experiments are designed to verify the multipath error mitigating performance of the BDS B1/B2 dual frequency MHM model, both in the observation domain and the coordinate domain. The basic principle of the multipath SD method and BDS dual-frequency positioning model are described in the section on the BDS dual-frequency precise positioning model. The spatial repetition characteristics of carrier-phase residuals and the BDS dual-frequency MHM model are built in the section on the MHM model for BDS dual-frequency carrier phase multipath mitigation. The experiments and analysis of these results is presented in the section on experiment analysis. A discussion and conclusions are in the section on conclusions.

## 2. Dual-Frequency DD Residuals and Reconstruction of SD Residuals

In the process of relative positioning, ionospheric errors, tropospheric errors and receiver clock errors are eliminated by means of a double difference combination model. Generally, the remaining DD residuals are regarded as multipath errors, but considering that the reference satellite will change with time, the DD residuals are converted to single-error residuals. In this section, the double frequency observation equation of BDS is listed and the theoretical method of converting the DD residue to the SD residue is derived.

When BDS is used for deformation monitoring, the double difference (DD) observation equation is generally used to eliminate the receiver and satellite error. The multipath error is highly related to the monitoring environment, and it cannot be eliminated or mitigated by constructing the double difference observation equation. Assuming $\nabla \Delta {\left(\cdot \right)}_{br}^{ij}=\Delta {\left(\cdot \right)}_{br}^{j}-\Delta {\left(\cdot \right)}_{br}^{i}$ as the DD operator, the BDS DD observation equation in a short baseline (less than 10 km) can be described as follows:

(1) $$\begin{array}{cc} \nabla ∆p_{br,f}^{ij} & =\nabla ∆{\hat{\rho }}_{br}^{ij}+\nabla ∆u_{br}^{ij}\cdot ∆r_{r}^{}+\nabla ∆CM_{br,f}^{ij}+\nabla ∆\varepsilon_{code,br,f}^{ij}\\ \lambda_{f}\cdot \nabla ∆\phi_{br,f}^{ij} & =\nabla ∆{\hat{\rho }}_{br}^{ij}+\nabla ∆u_{br}^{ij}\cdot ∆r_{r}^{}+\lambda_{f}\cdot \nabla ∆N_{br,f}^{ij}+\nabla ∆PM_{br,f}^{ij}+\nabla ∆\varepsilon_{phase,br,f}^{ij} \end{array}$$

where, i and j are the BDS satellite PRN, b and r represent the base station and the rover station, $\Delta r_{r}$ is the receiver coordinates changed positive, $\nabla \Delta u_{br}^{ij}$ is the coefficient of the state matrix, f is the frequency ID, $\lambda_{f}$ represent the wavelength of frequency *f*, $\nabla \Delta p_{br,f}^{ij}$ and $\nabla \Delta \phi_{br,f}^{ij}$ are the DD pseudo-range and carrier phase, $\rho_{r}^{j}$ is the DD range on the time of single sending from satellite, $CM_{br,f}^{ij}$ and $PM_{br,f}^{ij}$ are the pseudo-range and carrier phase multipath error of frequency f, $\varepsilon_{code,br,f}^{ij}$ and $\varepsilon_{phase,br,f}^{ij}$ are the pseudo-range and carrier phase observation error on the frequency f, and $\nabla \Delta N_{br,f}^{ij}$ is the DD ambiguity.

In relative positioning, the satellite with the highest elevation angle is generally selected as the reference satellite, then the DD observation equations are formed between other satellites and the reference satellite. Given that the satellite orbit of BDS is heterogeneous and the reference satellite changes frequently, it is difficult to correctly reflect the multipath error in the DD carrier phase residual. It is necessary to convert the DD residual into the SD residual [22].

Assume that there are n common-view satellites between the reference and the rover station, the reference satellite is marked as i, and the others were marked as 1~⁡n−1. The residual of the dual-frequency DD observation equation can be expressed as:

(2) $$\begin{array}{l} \left[\begin{array}{l} dd_{f_{1}}\\ dd_{f_{2}} \end{array}\right]=\left[\begin{array}{l} B_{f_{1}}\\ B_{f_{2}} \end{array}\right]\left[\begin{array}{l} s_{X}\\ s_{Y}\\ s_{Z} \end{array}\right]+\left[\begin{array}{l} A_{f1}\\ A_{f_{2}} \end{array}\right]\left[\begin{array}{l} a_{f_{1}}\\ a_{f_{2}} \end{array}\right]-\left[\begin{array}{l} L_{f_{1}}\\ L_{f_{2}} \end{array}\right]\\ dd_{f_{1}}={\left[\begin{array}{c} dd_{{{}_{br}}_{,f_{1}}}^{i,1}\\ \vdots \\ dd_{{}_{{}_{br,f_{1}}}}^{i,n-1} \end{array}\right]}_{(n-1)/2,1}dd_{f_{2}}={\left[\begin{array}{c} dd_{{{}_{br}}_{,f_{2}}}^{i,1}\\ \vdots \\ dd_{{}_{{}_{br,f_{2}}}}^{i,n-1} \end{array}\right]}_{(n-1)/2,1} \end{array}$$

The conversion relationship between dual-frequency SD residual and dual-frequency double difference residual can be expressed as:

(3) $$\begin{array}{l} \left[\begin{array}{l} dd_{(n-1)/2,1,f1}\\ dd_{(n-1)/2,1,f2} \end{array}\right]=\left[\begin{array}{l} D_{f1}\\ D_{f2} \end{array}\right]\cdot \left[\begin{array}{l} sd_{n/2,1,f1}\\ sd_{n/2,1,f2} \end{array}\right]\\ D_{f1}=D_{f2}={\left[\begin{array}{ccccc} 1 & -1 & 0 & \ldots & 0\\ 1 & 0 & -1 & \ldots & 0\\ \vdots & \vdots & \vdots & \ddots & \vdots \\ 1 & 0 & 0 & \ldots & -1 \end{array}\right]}_{(n-1)/2,n} \end{array}$$

If we can reconstruct SD residuals from DD residuals using the transition matrix which contains the “zero mean” assumption $\sum w_{i}\cdot sd_{i}=0,w_{i}=\sin^{2}(el_{i})$, then the reconstructed SD residuals can be expressed as [22,37]:

(4) $${\left[\begin{array}{c} 0\\ dd_{br}^{i,1}\\ M\\ dd_{br}^{(n-1)/2,1} \end{array}\right]}_{n/2,1,f_{k}}={\left[\begin{array}{ccccc} w_{1} & w_{2} & w_{3} & \ldots & w_{n/2}\\ 1 & -1 & 0 & \ldots & 0\\ 1 & 0 & -1 & \ldots & 0\\ \vdots & \vdots & \vdots & \ddots & \vdots \\ 1 & 0 & 0 & \ldots & -1 \end{array}\right]}_{n/2,n/2,f_{k}}{\left[\begin{array}{c} sd_{br}^{1}\\ sd_{br}^{2}\\ \vdots \\ sd_{br}^{n/2} \end{array}\right]}_{n,1,f_{k}},f_{k}=f_{1},f_{2}$$

where ${sd}_{br}^{l}$ and ${dd}_{br}^{il}$ represent reconstructed SD residuals and DD residuals, respectively, and $w_{i}$ is the weighting factor. Since we use the weighting model of elevation angles, $w_{i}=\sin^{2}\left(\theta \right.)$ here.

After the above derivation, DD residuals can be converted to SD residuals. At this stage, the SD residue contains multipath error and measurement noise, and it is generally considered that the low-frequency component of the SD residual is the multipath error.

## 3. MHM Model for BDS Dual-Frequency Carrier Phase Multipath Mitigation

The multipath effect refers to that the signal transmitted by the satellite being reflected by the surrounding environment of the receiver, causing the actual observed value received by the receiver to deviate from the actual observed value transmitted by the satellite. In the process of signal transmission, the GNSS positioning accuracy can be greatly improved by mitigating the multipath effect as much as possible.

### 3.1. Multipath Basic Principle

The path deviation of satellite signals caused by multipath effects can be expressed as:

(5) $$S_{multi}=\frac{∆\varphi_{m}}{2\pi }\lambda =\frac{\lambda }{2\pi }\arctan(\frac{\alpha \sin(\frac{4\pi D}{\lambda }\sin\theta )}{1+\alpha \cos(\frac{4\pi D}{\lambda }\sin\theta )})$$

where *D* represents the horizontal distance between the receiver antenna and the reflector around the antenna, *λ* and *θ* are respectively the wavelength and the incidence angle of the reflected signal, *α* is the reflection coefficient of the reflector, and $\Delta \varphi_{m}$ represents the phase delay caused by multipath effects. We can find that the multipath errors are mostly affected by horizontal distance (*D*), incident angle (*θ*) and reflection coefficient (*α*). The reflective surface, antenna and their properties remain unchanged in most cases of deformation monitoring, which means that the horizontal distance and reflection coefficient are constant. Hence it is only the incidence angle of reflected signals that could change multipath errors. Meanwhile, the incidence angle of reflected signals is determined by the position of satellites in the sky which periodically repeats in specific orbits. This indicates that we can mitigate multipath effects for current data by subtracting the multipath error models extracted from the last period data.

### 3.2. Spatial Repetition Characteristics Analysis of BDS Carrier Phase Observation Residuals

When the environment around the monitoring station is static, the multipath error is highly correlated with the incidence angle of the satellite signal, and the variation characteristics of the satellite signal incidence angle are consistent in the spatial domain. However, the variation characteristics of different satellites orbit types or different observation frequencies vary. Taking the carrier phase observations of different frequencies and different satellites as the focus, the relationship between DD residual and SD of carrier phase observation is constructed, and the carrier phase SD residual is obtained [26,28]. The variation characteristics of the SD error residuals can be expressed by establishing a MHM multipath error model with the elevation angle and azimuth angle as grid points.

**Figure 1:**
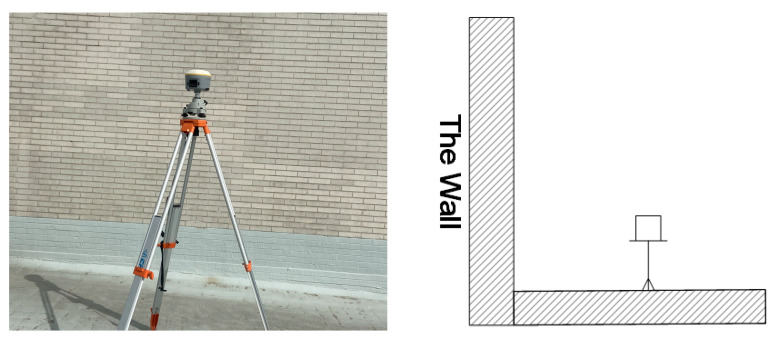
Observation environment around the rover station.

To verify the repetition characteristics of BDS multipath error in the spatial domain, a set of BDS data were collected from 27 September to 10 October 2017 at a frequency of 1 Hz and using 5° as the cutoff elevation angle at the roof of the SESI building, China University of Mining and Technology, as shown in Figure 1. The two GNSS receivers are Trimble R10 units. One of them was set in an unobstructed environment as a base station while another one was placed about 5 m away from a white wall as the rover station. The length of the baseline is 62.210 m. To ensure that the base station was not affected by multipath noise and the rover station had an obvious multipath effect, the anti-multipath function of the rover station was switched off while the same function of the base station was switched on.

**Figure 2:**
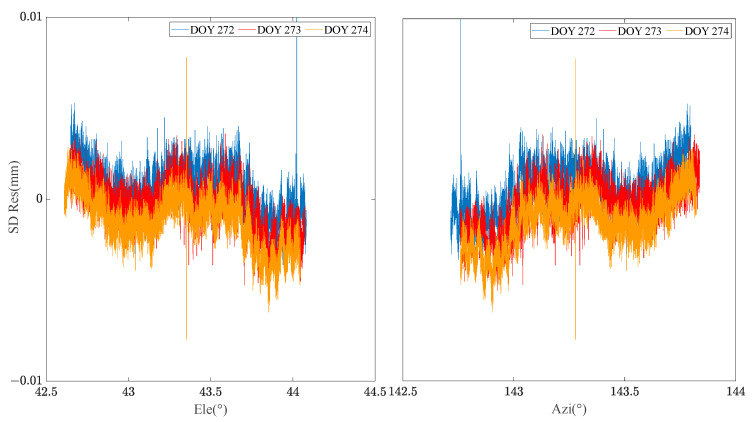
Relationship between multi-day MEO C12 SD residual and Ele/Azi.

**Figure 3:**
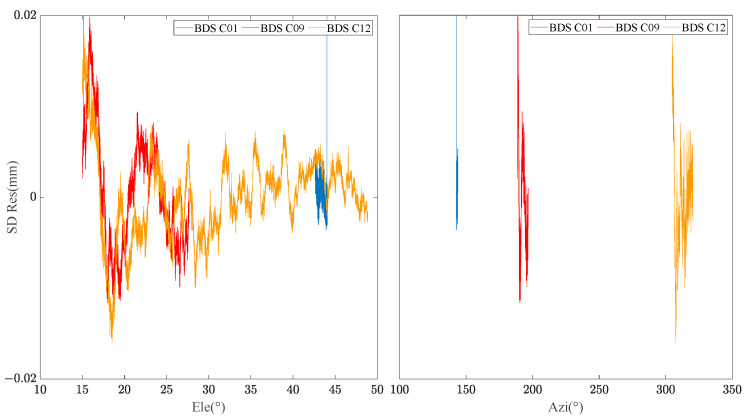
Relationship between C01/C09/C12 SD residual and Ele/Azi.

**Figure 4:**
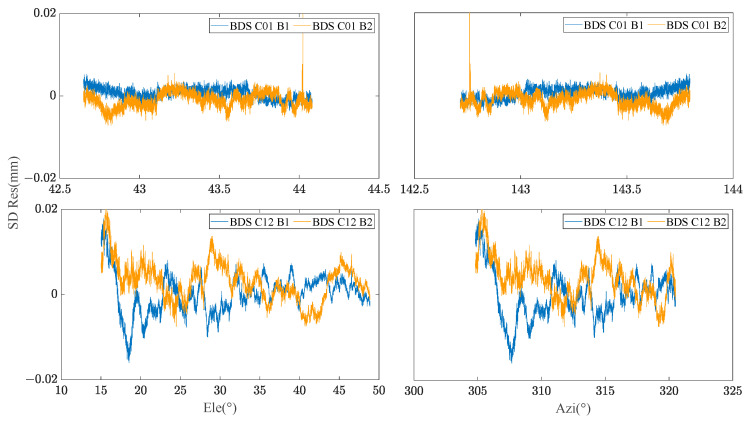
Relationship between C01/C12 B1/B2 SD residual and Ele/Azi.

By extracting the SD residuals of the B1 frequency carrier observations of BDS MEO C12 satellites for many days, the relationship between the SD residuals and elevation/azimuth angle was constructed respectively. Figure 2 demonstrates that the SD residuals of carrier phase observations in adjacent days of the same satellite are highly correlated with the spatial domain distribution composed of elevation and azimuth angle. Figure 3 manifests the relationship between the satellite’s spatial domain distribution and the SD residuals of carrier phase of different satellites in the same day. It can be found that the SD residuals of different satellites are not related in the spatial domain distribution composed of elevation and azimuth angle. Figure 4 shows the relationship between the satellite’s spatial domain distribution and the SD residuals of carrier phase of different frequencies for the same satellite. It indicates that there is rarely correlation between the SD residuals of carrier phase of different frequencies.

### 3.3. Methodology of BDS Dual-Frequency MHM

Based on the above characteristics in the spatial domain of the observations, taking the carrier phase SD residuals of different frequencies and various satellites as the research focus, an MHM model is constructed with the receiver as the coordinate origin and the azimuth and elevation angles as the longitude and latitude. The model is mainly divided into two parts: the hemispherical geometric model and the hemispherical grid parameter fitting.

#### 3.3.1. Hemispherical Geometric Model

**Figure 5:**
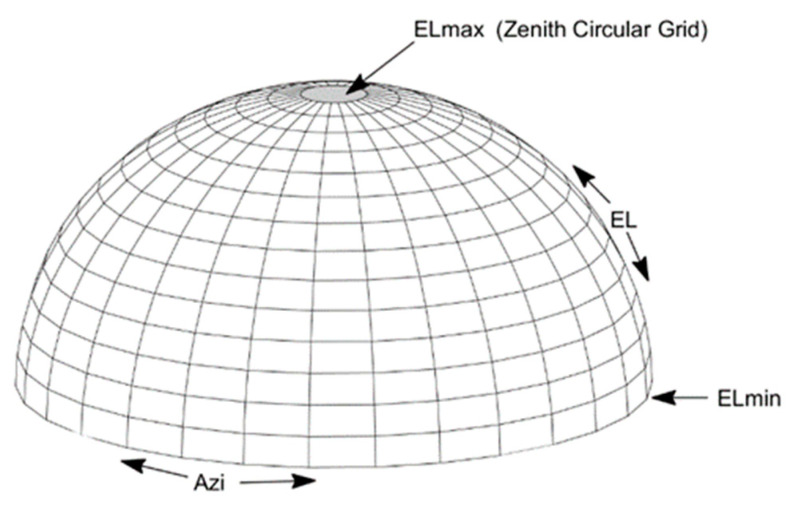
Schematic of HGM.

The SD residual variation in the BDS carrier phase observations is related to the satellites’ azimuth, elevation angles, and BDS frequency. The Multipath Hemispherical grid is constructed with the receiver as the coordinate origin and the azimuth and elevation angles as the longitude and latitude. Assuming that the min elevation angle is $EL_{min}$, the max elevation angle ($EL_{max}$) is less than 90 degrees, and the range of the azimuth (Azi) is from 0–360 degrees, the Zenith circular grid can be constructed. The Hemispherical Geometric Model (HGM) can be divided into many grids based on the min latitude grid $d_{ele}$ and longitude grid $d_{azi}$. The schematic representation of HGM can be seen in Figure 5.

**Figure 6:**
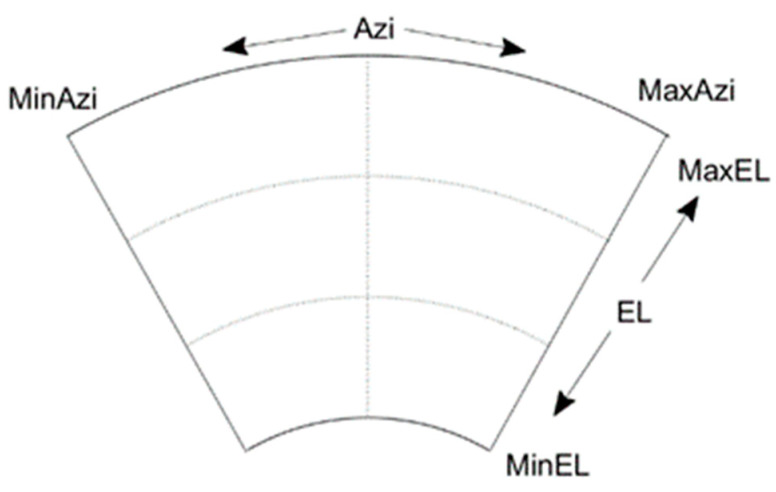
Schematic of BDS GEO HGM.

Due to the BDS GEO satellites operating on a small range orbit, the common hemispherical grid dividing method is too large for GEO satellites. It is necessary to construct a special and refined grid model for GEO satellites. We can obtain the minimum elevation angle $ELGEO_{min}$, maximum elevation angle $ELGEO_{min}$, the minimum azimuth angle $ELGEO_{min}$ and the maximum azimuth angle $ELGEO_{min}$ of the GEO satellites relative to the receiver by calculating the position of the GEO satellite in the zenith orbit. Then, the refined geometric model for GEO satellites can be constructed using the minimum latitudinal interval $dGEO_{ele}$ and the longitude interval $dGEO_{azi}$. The schematic representation of the GEO hemispherical geometric model, $HGM_{GEO}$ is shown in Figure 6.

#### 3.3.2. Hemispherical Grid Parameter Fitting

We refer to the data used in modeling as first-phase data and the corrected data as second-phase data. Since the repetition cycle of a BDS MEO satellite is 7 days, the data of 7 consecutive days are used as the first-phase data, and the data of the eighth day, that is, the data to be corrected by multipath, are used as the second-phase data.

Firstly, the wavelet analysis method is used to denoise the SD residuals between the stations obtained from the first-phase data solution, and the multipath errors of each satellite operating in each grid are extracted, which are distinguished by azimuth angle and elevation angle. Among them, only the SD residuals between satellite stations at the successful time of fixed ambiguity are extracted. The mesh parameters of the MHM geometric model were fitted with the multipath errors extracted from the first-phase data. The parameters in each grid are distinguished according to the satellites PRN and satellite frequency. Assuming that this is satellite i in grid a, the grid parameters can be described as:

(6) $$\begin{array}{l} Value_{a,i,f1}=\frac{\sum_{k=1}^{n}sd_{i,f1,k}}{n}\\ Value_{a,i,f2}=\frac{\sum_{k=1}^{n}sd_{i,f2,k}}{n} \end{array}$$

where, $sd_{i,f1}$ is the SD residuals of satellite i at the frequency f1; $sd_{i,f2}$ the SD residuals of satellite i at the frequency f2; and n is the number of SD residuals of the i satellite in grid a. The hemispherical grid parameter of each grid can be obtained by calculating the mean value of all SD residuals of satellite i in the grid.

**Figure 7:**
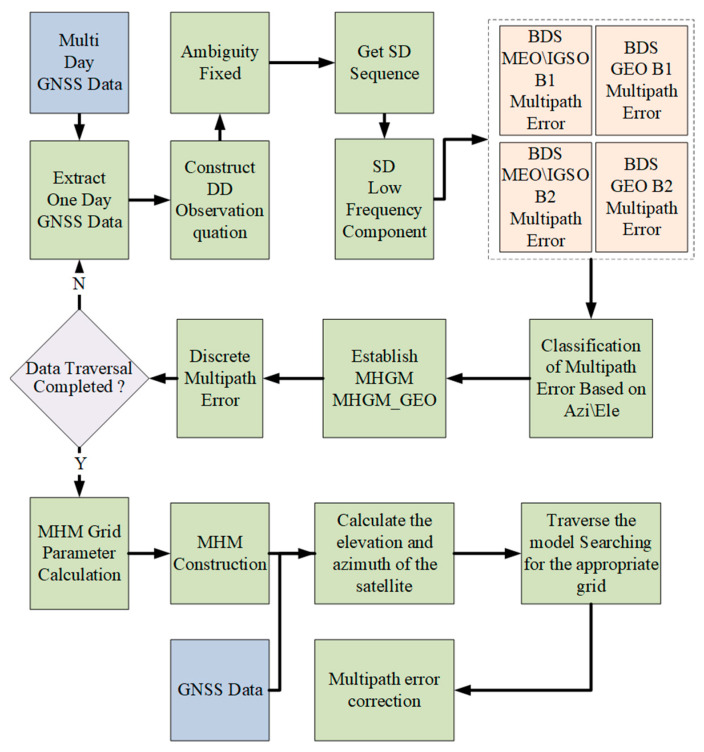
BDS dual frequency MHM and multipath correction flow chart.

In summary, the construction flow chart of the BDS dual frequency MHM is given in Figure 7. Firstly, the multi-day GNSS monitoring data were used as the first-phase data to fix the ambiguity rapidly. Based on the hypothesis of “zero mean”, the DD residuals were converted into SD residuals. Then, the low-frequency component of the SD residuals sequence of each satellite was extracted by wavelet analysis method as the multipath error. Secondly, a hemisphere model based on elevation and azimuth is established based on the monitoring station as the origin, and the multipath errors of each satellite in the first-phase data are used to fit the mesh parameters of each MHM. In the process of multipath error correction, all grids of the MHM model are traversed based on the elevation and azimuth angles of the satellite until the corresponding model values are found, and then the station single-differenced residuals of the satellite are corrected. Establishing observation equations based on the above process, followed by baseline calculation, can maximize the mitigation of multipath effects in carrier phase observations.

It is worth mentioning that this method can also be applied to medium-to-long baselines and Network RTK [38]. For example, in Network RTK, accurately calculating the ambiguity, tropospheric delay and ionospheric delay between reference stations can extract multipath errors, establish MHM models to mitigate multipath effects and improve the interpolation accuracy of atmospheric errors.

## 4. Experiment Analysis

In order to verify the effectiveness of the proposed method, the multipath simulation experiment of CUMT building roof (Figure 1) and actual monitoring data of river culvert were processed respectively in this part, and different solutions were adopted to compare the single-error residual sequence and coordinate sequences of BDS GEO/IGSO/MEO satellites before and after MHM method correction.

### 4.1. CUMT Building Roof Experiment Case

**Figure 8:**
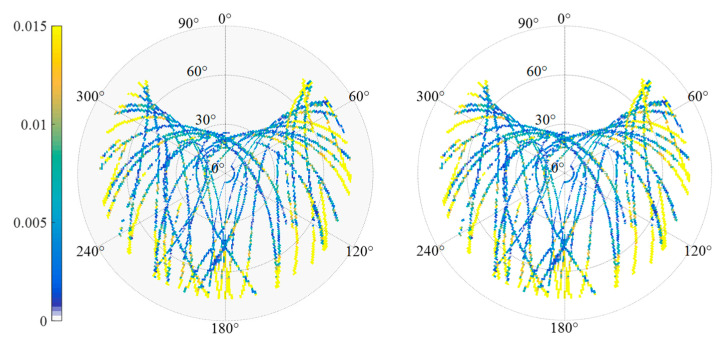
Multipath sky map of CUMT case ((**left**): B1 data, (**right**): B2 data).

The data set described in Section 3 is used to evaluate the performance of the BDS MHM method. Table 1 shows the configuration parameters of the MHM model for different BDS constellation satellites. The SD-MHM models established based on Table 1 are shown in Figure 8.

**Figure 9:**
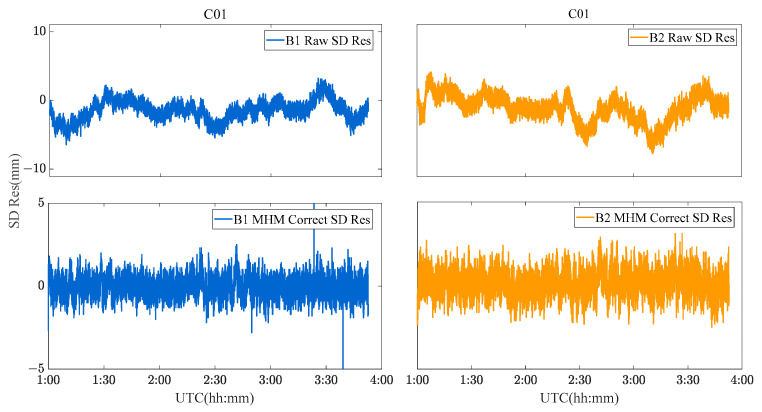
C01 SD residuals and their MHM mitigated series.

**Figure 10:**
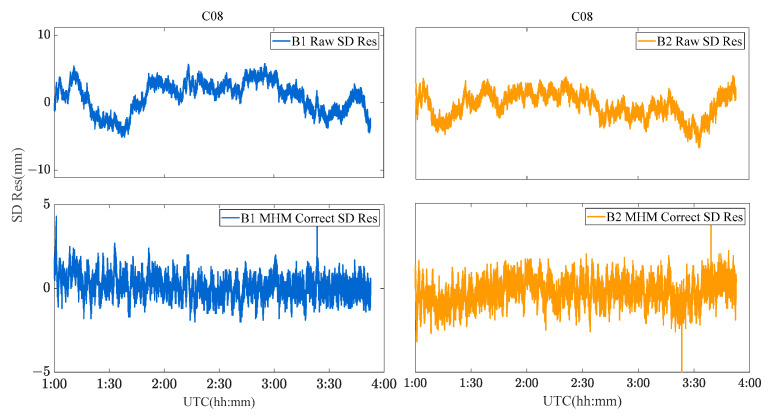
C08 SD residuals and their MHM mitigated series.

**Figure 11:**
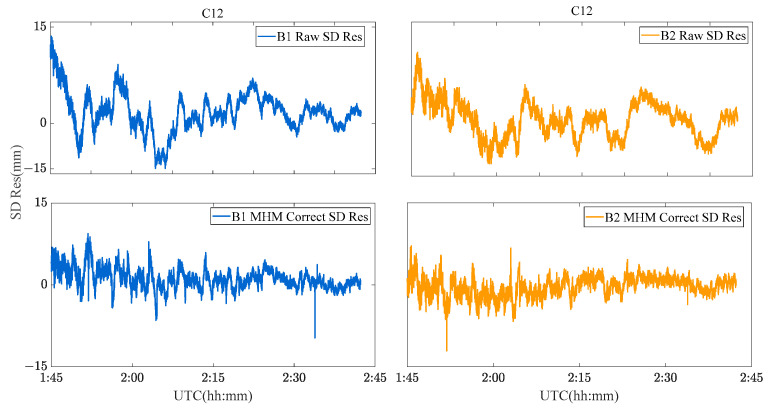
C12 SD residuals and their MHM mitigated series.

**Figure 12:**
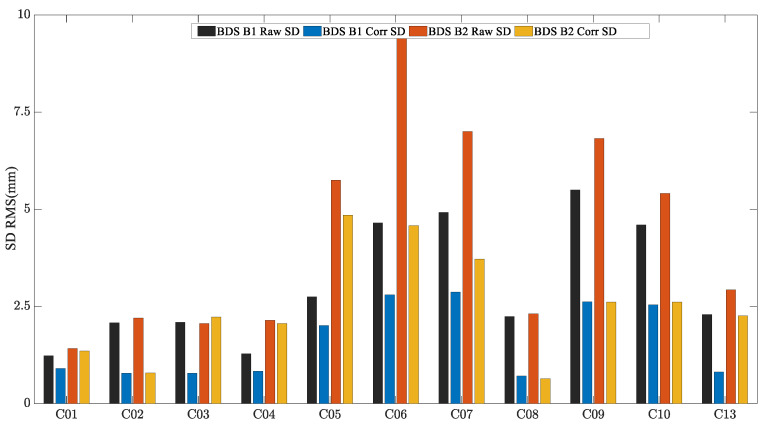
BDS SD residuals accuracy statistical chart of CUMT case.

Due to the limitation of the length of this paper, the performance of the MHM method of BDS GEO/IGSO/MEO satellites is discussed and analyzed by taking C01 (GEO), C08 (IGSO) and C12 (MEO) satellites as examples. Figure 9, Figure 10 and Figure 11 show the original SD residuals at both B1 and B2 frequencies and their mitigated series by MHM method. In terms of the multipath model trend, both are almost the same, and the series fluctuates at 0 after the mitigation. The statistical chart of different BDS satellites’ SD residuals is shown in Figure 12.

**Figure 13:**
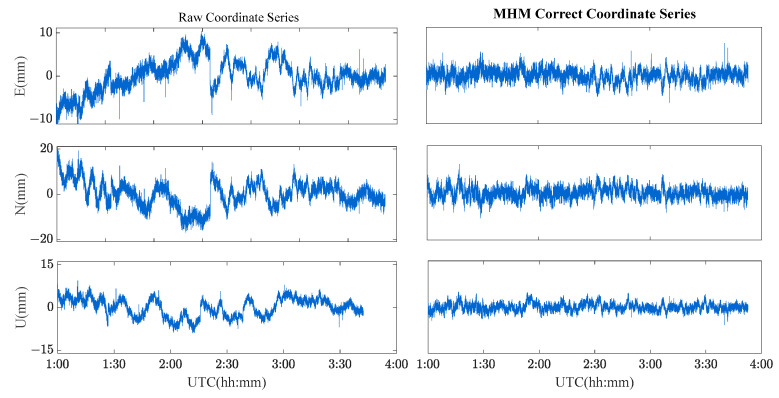
Original baseline series and MHM-corrected series for BDS single-frequency solutions.

**Figure 14:**
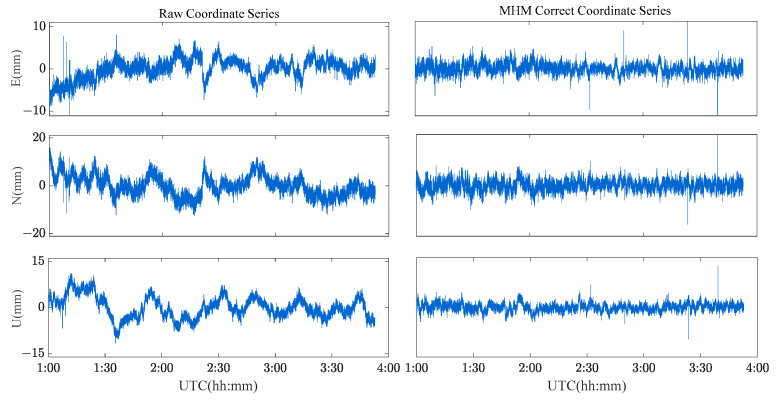
Original baseline series and MHM-corrected series for BDS dual-frequency solutions.

Four schemes were used to compare the performances of single-frequency/dual-frequency BDS multipath mitigation. These schemes are listed in Table 2. Figure 13 and Figure 14 show the solution sequence of the original baseline residuals and the corrected baseline residuals of the MHM method. The detailed statistical results are shown in Table 3. In Table 3, it can be found that multipathing affects the accuracy of the original baseline (scheme A and C), and after the multipathing effect is mitigated by the MHM method, the accuracy of the baseline in the ENU direction is improved, indicating that the MHM method can significantly mitigate multipathing in both single-frequency and dual-frequency cases. 

The single-frequency comparative experiment shows that the MHM method has a significant correction effect on the baseline sequence, the average improvement accuracy is 71%, and the average ambiguity fixed rate is increased by 4%. The dual-frequency comparative experiment shows that the MHM method also results in an obvious improvement for the baseline residuals, and the average improvement accuracy is 50%. The ambiguity success rate is 100%. By constructing the multipath error model, the baseline accuracy can be effectively improved, and the baseline sequence after multipath mitigation presents the characteristics of white noise.

### 4.2. River Culvert Deformation Monitoring Experiment Case

**Figure 15:**
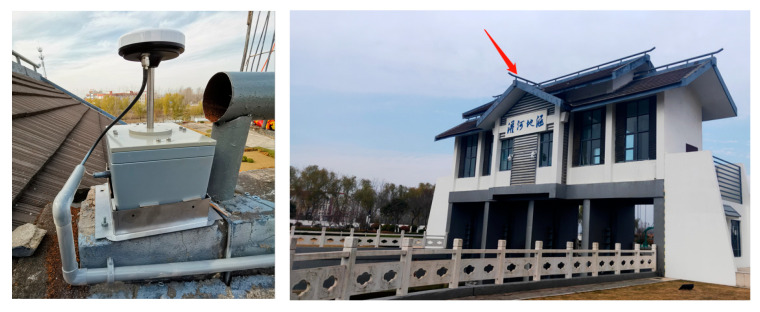
Environment of experiment.

**Figure 16:**
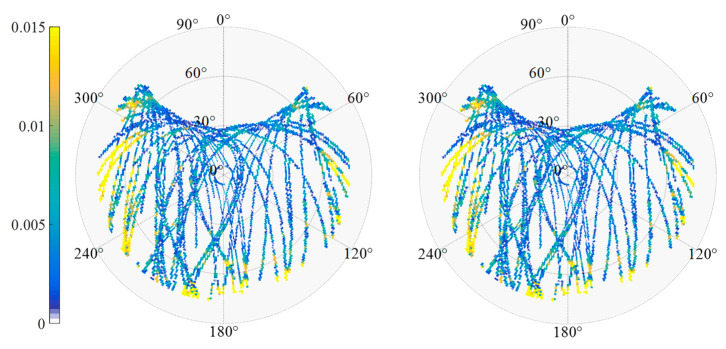
Multipath sky map and histogram of residual ((**left**): B1, (**right**): B2).

A GNSS monitoring receiver was set on the river culvert roof to monitor the building deformation caused by river confluence. In Figure 15, the red arrow is the set position of GNSS receiver antenna. The base GNSS station was set in the open-sky area around the culvert to collect the base station observation data. Data was collected for days of the year (DOY) 355–365 in 2020 for eleven consecutive days. The data sampling rate was 1 Hz, and the satellite elevation angle cutoff was 15°. As shown in Figure 16, most of the multipath error is due to the reflection of the north sloping roof.

The data on DOY 355–364 were collected to build the MHM, improve it, and extract the carrier phase residual sequences of all the satellites before and after multipath correction. The MHM models were used to mitigate the data on DOY 365 and obtain the refined baseline series to evaluate the MHM method performance.

**Figure 17:**
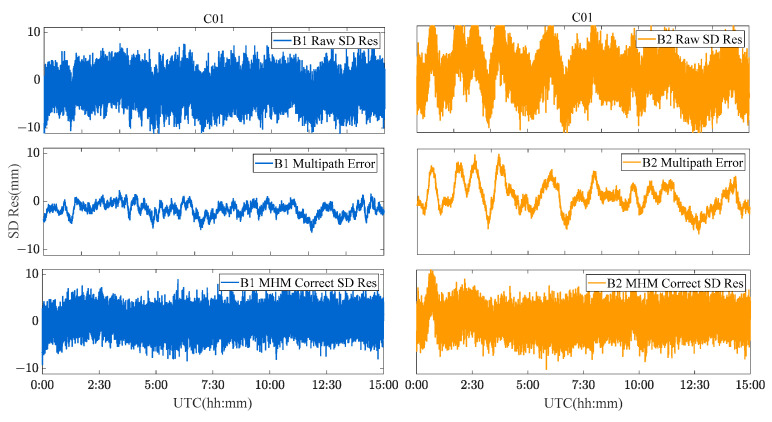
C01 SD residuals and their MHM-mitigated series for the river culvert experiment case.

**Figure 18:**
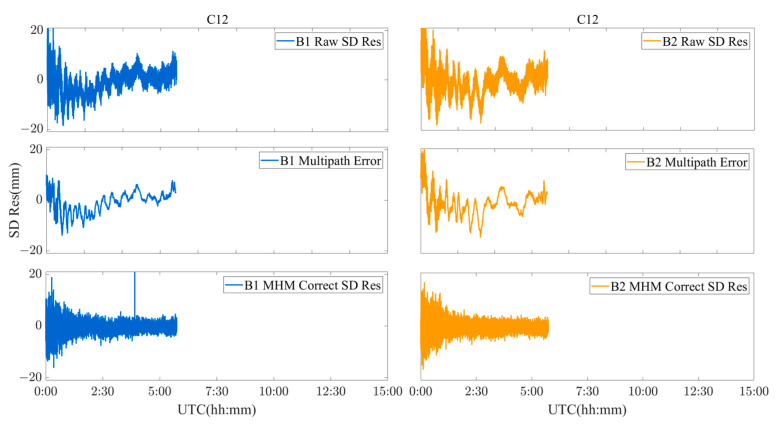
C12 SD residuals and their MHM-mitigated series for the river culvert experiment case.

**Figure 19:**
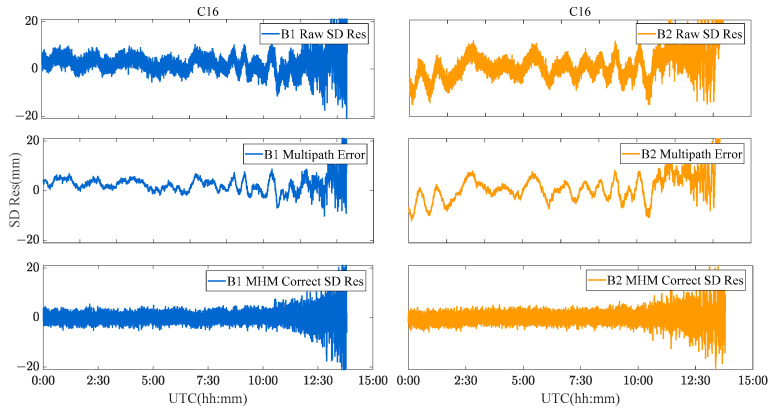
C16 SD residuals and their MHM-mitigated series for the river culvert experiment case.

**Figure 20:**
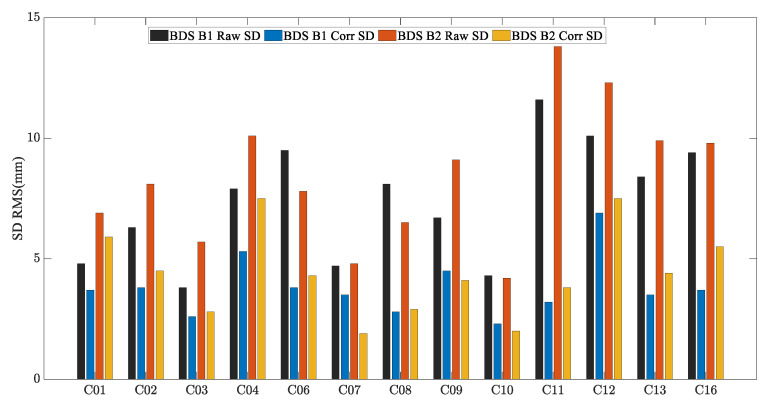
BDS SD residuals accuracy Statistical Chart for river culvert experiment Case.

To analyze the performance of the MHM method in a realistic deformation monitoring scenario, the C01, C12, and C16 satellites were used as an example of BDS GEO/IGSO/MEO satellites. Figure 17, Figure 18 and Figure 19 show the original SD residuals at both B1 and B2 frequencies and their mitigated series using the MHM method. It can be found that there are obvious low-frequency components in the original SD residual sequence of all satellites, and the trend of the low-frequency components is highly similar to the multipath error component extracted by the MHM method: the corrected SD residual sequence obtained from MHM eliminates the low-frequency component and shows characteristics of white noise. Figure 20 shows the SD residuals of BDS satellites’ B1/B2 frequency before and after correction by MHM method.

The same schemes were used to compare the performances of single-frequency/dual-frequency BDS multipath mitigation for real deformation monitoring scenarios, which can be found in Table 3. 

**Figure 21:**
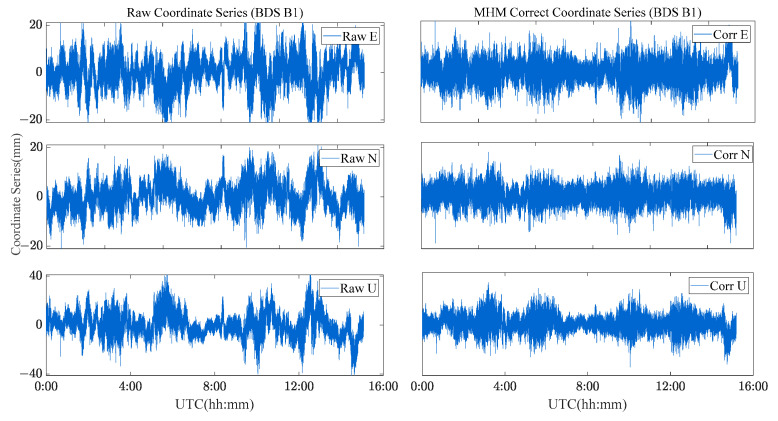
Original baseline series and MHM-corrected series of BDS single-frequency solutions for the river culvert experiment case.

**Figure 22:**
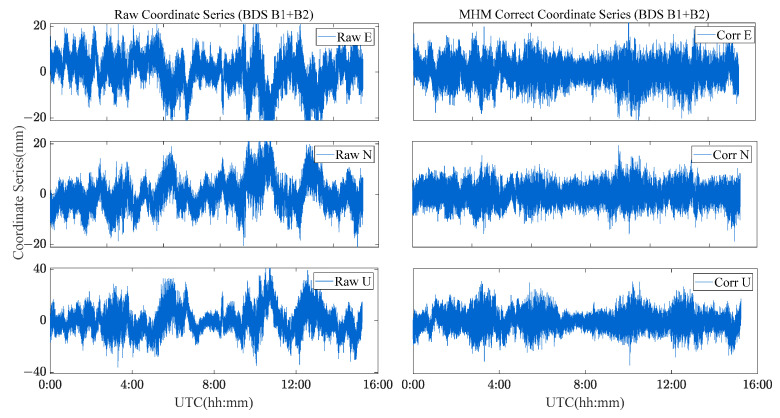
Original baseline series and MHM corrected series of BDS dual-frequency solutions for the river culvert experiment case.

Figure 21 and Figure 22 show the solution sequence of the original baseline residuals and the corrected baseline residuals of the MHM method for the river culvert deformation monitoring scenario. The detailed statistical results are shown in Table 4. We found that the multipath affects the accuracy of the original baseline (Scheme A and C), while the MHM method significantly mitigates the multipath error both for single-frequency and dual-frequency. 

The single-frequency comparative experiment shows that the MHM method has a significant correction effect on the baseline sequence, the average improvement accuracy is 83.3%, and the average ambiguity fixed rate is increased by 7%. The dual-frequency comparative experiment shows that the MHM method also results in obvious improvement for the baseline residuals, improving the accuracy by 57.2%. The ambiguity success rate is almost 99.6~99.8%. By constructing the multipath error model, the baseline accuracy can be effectively improved, and the baseline sequence after multipath mitigation presents the characteristics of white noise. The dual-frequency error EMS of the E, N, and U components are improved to 1.4 mm, 1.5 mm and 3.7 mm, respectively.

## 5. Conclusions

We proposed an SD residual-based multipath hemispherical map for the BDS dual-frequency carrier phase for dynamically mitigating the multipath errors in the dual-frequency carrier phase data for a real deformation monitoring scenario. In the proposed method, DD residuals are converted to SD residuals, and the multipath errors of each satellite are corrected respectively considering the differences between BDS satellites of different orbital types, which solves the problem that DD residuals change with the change in reference satellites. The developed method takes the elevation and the azimuth angles of the satellites as independent variables to construct the MHM model. The real scenario experiment results indicate that the MHM method has a significant correction effect on the baseline sequence: the average improvement accuracy is 83.3% and 57.2% for single-frequency and dual-frequency, respectively; the average ambiguity fixed rate is increased by 7% for single-frequency data; and the dual-frequency error EMS of E, N, and U components is improved to 1.4 mm, 1.5 mm and 3.7 mm, respectively.

## Figures and Tables

**Table 1:** MHM model configuration parameters.

Satellite System	Satellite Orbit	$dGEO_{ele}$	$dGEO_{azi}$
BDS	GEO	0.05°	0.05°
	IGSO	1°	1°
	MEO	1°	1°

**Table 2:** BDS solution schemes setting.

Solution	Satellite System	Frequency	Multipath Model	Cycle Slip Detection Method
Scheme A	BDS	B1	--	Pseudo-range/carrier combination method
Scheme B			MHM	
Scheme C		B1 + B2	--	TurboEdit
Scheme D			MHM	

**Table 3:** Detailed statistical results for different schemes.

Control Group	Scheme	RMS/mm			Fixed Rate
		E Direction	N Direction	U Direction	
Single Frequency	A	2.6	4.2	8.9	94.0%
	B	0.8 (69.2% ↑)	1.0 (76.2% ↑)	2.8 (68.5% ↑)	99.9%
Dual Frequency	C	1.2	2.1	5.1	100%
	D	0.7 (41.7% ↑)	0.9 (57.1% ↑)	2.4 (52.3% ↑)	100%

↑ is the percentage improvement in accuracy relative to before correction.

**Table 4:** The detail statistical results for different scheme for the river culvert experiment case.

Control Group	Scheme	RMS/mm			Fixed Rate
		E Direction	N Direction	U Direction	
Single Frequency	A	8.2	9.0	23.9	92.2%
	B	1.4 (82.9% ↑)	1.6 (82.2% ↑)	3.6 (84.9% ↑)	99.6%
Dual Frequency	C	2.8	3.5	10.4	99.6%
	D	1.4 (50.0% ↑)	1.5 (57.1% ↑)	3.7 (64.4% ↑)	99.8%

↑ is the percentage improvement in accuracy relative to before correction.

## Data Availability

Not applicable.
